# AI-Powered Adaptive Disability Prediction and Healthcare Analytics Using Smart Technologies

**DOI:** 10.3390/diagnostics15162104

**Published:** 2025-08-21

**Authors:** Malak Alamri, Mamoona Humayun, Khalid Haseeb, Naveed Abbas, Naeem Ramzan

**Affiliations:** 1 Department of Computer Science, College of Computer and Information Sciences, Jouf University, Sakaka 72311, Saudi Arabia; mzalamri@ju.edu.sa; 2 Research and Innovation Groups, King Salman Center for Disability Research, Riyadh 11614, Saudi Arabia; 3 Department of Computing, School of Arts Humanities and Social Sciences, University of Roehampton, London SW15 5PH, UK; 4 Department of Computer Science, Islamia College Peshawar, Peshawar 25120, Pakistan; khalid.haseeb@icp.edu.pk (K.H.); naveed.abbas@icp.edu.pk (N.A.); 5 School of Computing, Engineering and Physical Sciences, University of West of Scotland, Paisley PA1 2BE, UK; naeem.ramzan@uws.ac.uk

**Keywords:** disease diagnosis, healthcare system, artificial intelligence, wearable sensors, edge computing

## Abstract

**Background**: By leveraging advanced wireless technologies, Healthcare Industry 5.0 promotes the continuous monitoring of real-time medical acquisition from the physical environment. These systems help identify early diseases by collecting health records from patients’ bodies promptly using biosensors. The dynamic nature of medical devices not only enhances the data analysis in medical services and the prediction of chronic diseases, but also improves remote diagnostics with the latency-aware healthcare system. However, due to scalability and reliability limitations in data processing, most existing healthcare systems pose research challenges in the timely detection of personalized diseases, leading to inconsistent diagnoses, particularly when continuous monitoring is crucial. **Methods**: This work propose an adaptive and secure framework for disability identification using the Internet of Medical Things (IoMT), integrating edge computing and artificial intelligence. To achieve the shortest response time for medical decisions, the proposed framework explores lightweight edge computing processes that collect physiological and behavioral data using biosensors. Furthermore, it offers a trusted mechanism using decentralized strategies to protect big data analytics from malicious activities and increase authentic access to sensitive medical data. Lastly, it provides personalized healthcare interventions while monitoring healthcare applications using realistic health records, thereby enhancing the system’s ability to identify diseases associated with chronic conditions. **Results**: The proposed framework is tested using simulations, and the results indicate the high accuracy of the healthcare system in detecting disabilities at the edges, while enhancing the prompt response of the cloud server and guaranteeing the security of medical data through lightweight encryption methods and federated learning techniques. **Conclusions**: The proposed framework offers a secure and efficient solution for identifying disabilities in healthcare systems by leveraging IoMT, edge computing, and AI. It addresses critical challenges in real-time disease monitoring, enhancing diagnostic accuracy and ensuring the protection of sensitive medical data.

## 1. Introduction

In recent decades, healthcare systems have undergone rapid growth to facilitate remote diagnosis and timely medical treatments [1,2,3]. These systems primarily rely on the Internet of Things and future wireless technologies for seamless connectivity, enabling long-term communication with connected devices [4,5]. The IoT-based Healthcare Industry 5.0 offers remarkable development, enhancing real-time monitoring and operational efficiency with the support and integration of innovative 5G/6G technologies [6,7]. On the other hand, edge computing [8,9] is a cutting-edge technology with various applications and enticing features, including inexpensive implementation and data transmission costs, unrestricted network access, and self-contained, long-term operation [10,11,12]. Additionally, IoT systems have emerged as a critical paradigm, enhancing real-time health data collection and facilitating their processing closer to data sources [13,14]. Such a communication system not only reduces latency but also improves efficiency and data management with effective resource utilization in smart cities. It provides efficient and real-time healthcare monitoring, improving community services in crucial areas. The continued monitoring and analysis of medical records increases the possibility of early disability detection and provides timely treatment with personalized care [15,16,17]. However, healthcare applications require optimized and trusted processing under critical environments with minimized bandwidth consumption to ensure privacy-preserving and fault-tolerant systems. Moreover, the uncertain events of IoT networks make them vulnerable to communication threats and increases the possibility of disruption in health services [18,19]. In addition, the need for robust and scalable infrastructure in Healthcare Industry 5.0 demands data privacy and ethical considerations in processing medical requests and diagnosing diseases over the insecure Internet [20,21,22]. Our research aims to develop a framework for a healthcare system that leverages edge computing to facilitate timely decision-making in identifying disabilities and securing patient records in an unpredictable wireless environment. The following are the main contributions of the proposed framework.

- It develops a scalable AI-driven disability monitoring system that utilizes health sensors to collect physiological and behavioral data, ensuring latency and rapid response through local-level edge processing.
- It explores federated learning techniques to protect health records while ensuring compliance with privacy policies and enhancing trust in big data analytics for medical services.
- It utilizes a lightweight intelligent algorithm to observe health metrics, provide personalized healthcare and timely interventions, and offer adaptive solutions for real-time data analysis.

The remainder of the paper is structured as follows: Section 2 discusses related works. Section 3 highlights the main contributions and the algorithms in detail. Section 4 explains the performance results. Finally, Section 5 concludes this research work.

## 2. Related Work

The IoT-based network integrated with healthcare systems has shown significant development, especially in detecting human diseases based on real-time data [23,24]. These systems capture a vast amount of health data from patients’ bodies, process it, and analyze it to inform decision-making and take action. Many artificial intelligence and machine learning models have been explored to train the model and facilitate the process of early disease detection [25,26]. Despite these advancements, several research challenges still exist while coping with big data and generating an accurate disease identification mechanism promptly [27,28,29]. Moreover, security, along with trusted communication, is another issue in the design of the healthcare system under diverse conditions [30,31]. The authors of [32] proposed a secure and scalable framework for healthcare data transmission in IoT networks, featuring an optimized routing protocol. Medical data is collected using healthcare devices and preprocessing techniques to clean the raw data. K-nearest neighbor (KNN) imputation is performed, and principal component analysis (PCA) is used to reduce the data’s dimensionality. The features are extracted from the preprocessed data using modified local binary patterns (MLBPs). The proposed fuzzy dynamic trust-based RPL (FDT-RPL) protocol enhances data security in low-power and lossy networks by integrating a fuzzy dynamic trust-based RPL algorithm with the Butter Ant Optimization (BAO) algorithm. In [33], the authors have proposed FC-SEEDA, a Fog Computing-based Secure and Energy-Efficient Data Aggregation scheme for Internet of Healthcare Things. The primary objective of the proposed work is to reduce energy consumption and communication overheads in healthcare applications by leveraging the distributed nature of fog computing. It also secures data aggregation between medical sensors and the cloud system, maintaining data protection. The authors proposed a combined approach with a feature-driven FCNN classification model [34] that improves the accuracy and efficiency of multimodal healthcare analysis. The proposed system comprehensively classifies neurological disorders using preprocessing and extracts features from voice signals and handwritten spiral images. The experimental results demonstrate its optimized routing efficiency, latency, and identification methods for real-time monitoring applications in the medical sector. In [35], the authors proposed a secure and energy-efficient data transmission framework for IoT-based healthcare (IoT-HC) systems, utilizing enhanced mayfly clustering-based Q-learning routing (EMCQLR) and exponential key-based elliptic curve cryptography (EKECC) techniques. Initially, double hash biometric-based authentication (DHABA) is employed to authenticate IoT users and prevent unauthorized network access. The cluster head selection is performed using the enhanced mayfly optimization algorithm (EMOA), and multiple clusters are formed for IoT sensors. To ensure reliable routing, duplication is first checked, followed by the application of the path-weighted Q reinforcement learning (PWQRL) model. In [36], the authors introduced the Healthcare System using Private Blockchain-based Cloud IoT (HSPBCI) services, which safeguards data from being compromised and ensures authorized access for IoT data. The main goal of the proposed framework is to secure block-based transactions, minimize encryption and decryption times, and maintain authorized access to health records. Only devices with the appropriate data keys are permitted to access patient data, ensuring data privacy.

### Problem Statement

Despite significant advancements in health monitoring, many existing approaches primarily rely on AI-driven techniques that focus on single-modal data. Additionally, most solutions overlook privacy concerns and lack trusted methods for processing sensitive medical data, limiting the accuracy of real-time monitoring systems and reducing confidence in early-stage disease detection, especially in disability identification. Furthermore, many proposed solutions fail to efficiently manage resources in healthcare service processing and decision-making, diminishing their impact on critical clinical applications. To address these challenges, we propose an optimized and reliable multi-phase healthcare framework that enhances the accuracy of disability detection by incorporating multi-modal data and trust-aware wireless computing collaboration. This framework aims to support early-stage detection and improve resource management in clinical settings, particularly for disabilities related to mobility and cognitive impairments, such as walking difficulties, balance issues, and sensory processing disorders, while ensuring enhanced security against potential threats.

## 3. Materials and Methods

**Figure 1:**
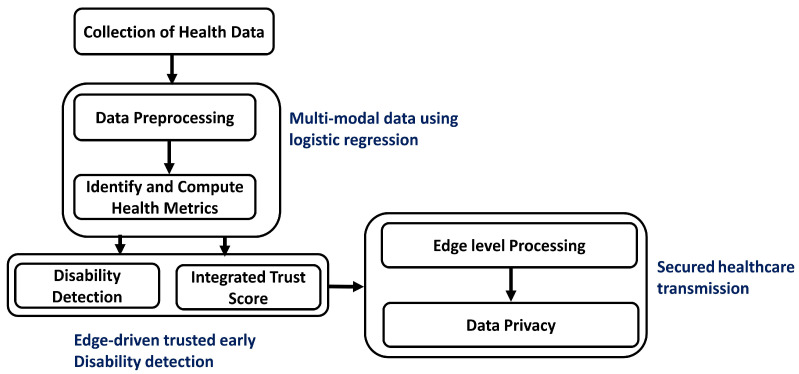
Developed stages of the proposed framework for the healthcare system with trusted edges.

In this section, we provide a comprehensive discussion on the design and development of the proposed framework, which leverages edge-AI technology for an autonomous disability detection system. The proposed framework comprises various states to identify the disabilities using wearable sensors integrated with intelligence and trusted computing, as depicted in Figure 1. It integrates different layers to interact with each other, identifying disabilities and combining edge-driven trust computing to achieve data privacy in an insecure wireless environment. Moreover, a multi-factor analysis is conducted, supported by a logistic regression model, to predict disability diseases and reduce the likelihood of false-positive detection. The proposed framework minimizes computing power requirements on constrained health devices, while efficiently managing resources, and enhances the response time in decision-making strategies of the healthcare system.

### 3.1. Clinical Role of Biomarkers in Disability Detection

This section outlines the operational categories of disability in the context of our study and discusses the clinical relevance and validation of the biomarkers used for disability detection. Disability in this context is defined as impairments in physical, cognitive, or sensory functioning that significantly restrict an individual’s ability to perform daily activities. The operational definition aligns with widely accepted clinical frameworks for disability assessment, which provide standardized approaches to assessing disability across various domains. The categories of disability addressed in the proposed research framework include mobility impairments, such as difficulties with walking and balance; sensory impairments, including vision and hearing loss; and cognitive impairments, such as memory loss, attention deficits, and speech difficulties. These categories were selected due to their significant impact on the individual’s ability to function independently and their relevance in clinical healthcare settings for diagnosis, intervention, and patient care management. To enhance the accuracy of disability detection, our framework utilizes three biomarkers: heart rate variability (HRV), motion data (MD), and speech rate (SR). HRV is a well-established biomarker for assessing autonomic nervous system dysfunction, which is commonly observed in individuals with mobility and cognitive impairments. It is particularly useful for evaluating the health of the cardiovascular system and detecting autonomic dysfunction, which is often linked to conditions such as cardiovascular diseases and neurodegenerative disorders like Parkinson’s disease and stroke. HRV provides valuable insights into the autonomic regulation of the heart rate, which is influenced by the body’s response to stress, physical activity, and other factors that can affect mobility and cognitive function.

### 3.2. Discussion

**Figure 2:**
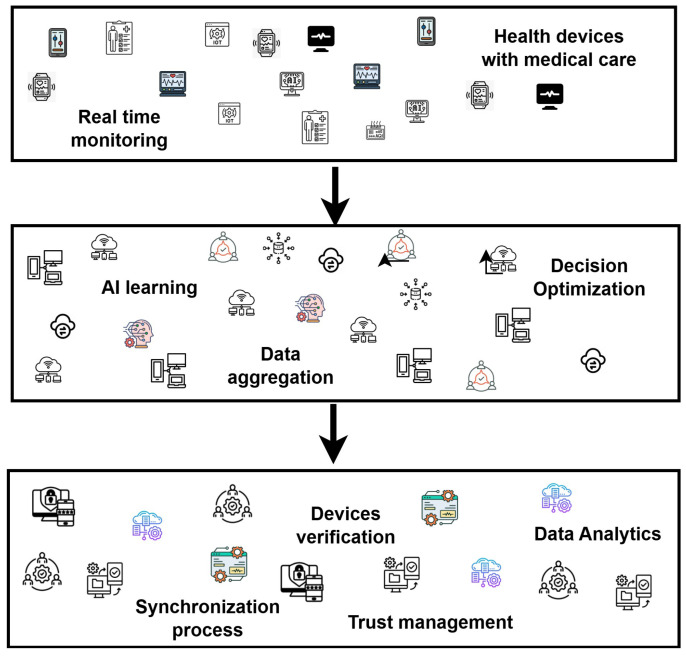
Layers of the proposed framework using trust with accurate diagnosis for a personalized healthcare system.

The proposed framework is composed of two main phases. Firstly, a scalable disability system is developed using real-time health metrics for regular patient monitoring, collecting behavioral data, and detecting early signs of disability disease. Moreover, medical data is processed locally using edge computing, resulting in a latency-aware healthcare system with minimal overhead for the health devices. In the second phase, robust and reliable security methods are applied to the medical data to protect it against threats and increase the trustworthiness of the healthcare system. Moreover, federated learning utilizes the patient’s data to be stored locally on health devices, eliminating the need to transmit it to centralized systems and thereby ensuring privacy and integrity. It aims to provide timely, personalized healthcare interventions by analyzing health metrics and ensuring the secure transmission of sensitive medical data through edge-level local processing, leveraging the collaboration of trusted IoT devices. The interaction of the layers in the proposed framework is illustrated in Figure 2.

Initially, IoMT sensors *s* collect the health records $x_{i,s}$ from the patient’s body, as specified in Equation (1), which are then further preprocessed to ensure the system’s accuracy. It comprises a normalization process, norm, applied to the selected features using the min−max scaling method, as defined in Equation (2).

(1) $$\mathrm{Sensor}\;\mathrm{Data}=\{x_{i,s}|1\leq i\leq n,\;1\leq s\leq m,\;x_{i,s}\in R\}$$

(2) $$x_{\mathrm{norm},i}=\frac{x_{i}-\min\left(x\right)}{\max(x)-\min(x)}\;\mathrm{for}\;i=1,2,\ldots ,n$$

The proposed framework offers a generalized computational method, utilizing Equation (3), and consistently determines various health metrics from real-time patient’ monitoring.

(3) $$\begin{array}{cc} X & =\sum_{i=1}^{n}f_{\mathrm{metric}}\left(x_{i},\theta \right)\\ \; & \;\mathrm{where}\;\theta =\{\mathrm{parameters}\} \end{array}$$

where *X* represents the set of health metrics, $f_{\mathrm{metric}}\left(x_{i},\theta \right)$ is a function that applies to sensor data $x_{i}$ at the collection point, and θ is related to a particular parameter in terms of weights or thresholds. The health metrics used in the proposed framework for analyzing patient conditions include heart rate variability (HRV) that indicates the difference between successive heart rate intervals, as defined in Equation (4); motion data (MD), which is utilized to determine physical abilities and movement patterns, based on the accelerometer data, as outlined in Equation (5); and speech rate (SR), which denotes the number of spoken words $W_{i}$ over the *i*-th time period, as denoted in Equation (6).

(4) $$\Delta RR_{i}=\left\{RR_{i+1}-RR_{i}|i=1,2,\ldots ,n\right\}$$

(5) $$∥a\left(t\right)∥=\sqrt{a_{x}^{2}+a_{y}^{2}+a_{z}^{2}}$$

where $a_{x}$, $a_{y}$, and $a_{z}$ represent the acceleration components in the *x*-, *y*-, and *z*-axes, respectively.

(6) $$SR=\frac{\sum_{i=1}^{n}T_{\alpha ,i}}{\sum_{i=1}^{n}W_{i}}$$

Based on the health metrics, our proposed framework utilizes logistic regression, a multi-class classifier, to identify disabilities, and explores the logistic function to map the input features with specific probabilities Prob as given in Equation (7).

(7) $$X=\left[\begin{array}{c} \mathrm{HRV}\\ \mathrm{MD}\\ \mathrm{SR} \end{array}\right]$$

### 3.3. Logistic Regression with Multi-Modal Data for Disability Prediction

In the proposed framework, logistic regression [37], a multi-class classifier, is employed to map the multi-modal feature vector X and identify the disability class $C_{k}$ using the sigmoid function for each class *k*, as shown in Equation (8). The logistic regression model computes the weighted sum of the features for each class, considering the multi-modal data, and then transforms it into a probability using the sigmoid function.

(8) $$P\left(C_{k}|X\right)=\frac{1}{1+e^{-z_{k}}}$$

(9) $$z_{k}=\sum_{m=1}^{M}\sum_{i=1}^{n_{m}}\beta_{imk}\cdot x_{im}$$

where *M* is the number of modalities (e.g., HRV, MD, SR), $n_{m}$ is the number of features in each modality, $\beta_{imk}$ is the learned parameter for each feature $x_{im}$, and $z_{k}$ is the weighted sum for class *k*. The framework incorporates edge computing to reduce response time and optimize the healthcare system’s resources. The extracted data from health metrics is processed locally at the edges by leveraging edge devices. This significantly enhances the system’s capability by enabling real-time disability detection and prompt forwarding for further analysis. The network edges use the logistic function for disability detection based on the extracted features, as shown in Equation (10).

(10) $$P\left(C_{k}|X\right)=\frac{1}{1+e^{-\sum_{m=1}^{M}\sum_{i=1}^{n_{m}}\beta_{imk}}x_{im}}$$

The final disability class prediction $\hat{C}$ is determined by selecting the class with the highest computed probability, as shown in Equation (11).

(11) $$\hat{C}=\left\{C_{k}|k=\arg\max_{k}P\left(C_{k}|X\right)\right\}$$

### 3.4. Security Aspects of Disability Prediction Using AI and IoMT

In the next stage, the proposed framework ensures the privacy of patient data using homomorphic encryption, as defined in Equation (12). This encryption technique allows medical computations to be performed on the encrypted data without the need for decryption, ensuring that sensitive medical data remains protected throughout the process. Homomorphic encryption guarantees high-level security for patient records while preserving their integrity, thereby maintaining data confidentiality throughout the system’s operation. In Equation (12), $E(x_{i})$ represents the encrypted value of each feature $x_{i}$ using homomorphic encryption, allowing computations to be performed securely without decrypting the data. This technique ensures privacy while enabling the efficient processing of patient data.

(12) $$P\left(C_{k}|E\left(X\right)\right)=\frac{1}{1+e^{-\sum_{i=1}^{n}\beta_{ik}E\left(x_{i}\right)}}$$

The framework also incorporates a trust model by using reputation scores to evaluate historical performance. Trust is continuously computed by assessing the probabilities of prediction failure ($F_{a}$) or success ($S_{u}$), as shown in Equation (13). This method ensures that disability predictions are made with high reliability in a secure environment, thus enhancing the trustworthiness of the entire healthcare system. In Equation (13), $T_{t}$ represents the trust score at time *t*. The trust score is updated dynamically based on the success or failure of predictions. The factor α controls how much influence the prediction success, $S_{u}$, has on updating the trust score, while the factor β adjusts the influence of prediction failure, $F_{u}$. The updated trust score $T_{t+1}$ is computed by adding the contribution of success and subtracting the contribution of failure, which reflects the system’s ability to make accurate predictions.

(13) $$T_{t+1}=T_{t}+\alpha \cdot S_{u}-\beta \cdot F_{u}$$

Moreover, the framework utilizes federated learning to train the system locally on individual IoMT devices, ensuring that sensitive health data remains confidential and is not transmitted externally. This approach eliminates the need for centralized data storage, further protecting health records. Federated learning ensures that health metrics are processed on the devices themselves, maintaining patient data privacy while still enabling collaborative learning across devices. This decentralized approach is critical for preserving the confidentiality of sensitive data, as shown in Equation (14).

(14) $$w_{k}^{t+1}=w_{k}^{t}-\eta \nabla L\left(w_{k}^{t}\right)$$

where $w_{k}^{t}$ represents the model weights at time *t*, and $w_{k}^{t+1}$ represents the updated model weights for class *k* at time t+1. The update process is performed locally on each IoMT device, ensuring that no sensitive data leaves the device. The learning rate, η, controls the step size for updating the weights, and the gradient $\nabla L(w_{k}^{t})$ is used to guide the update based on the model’s current performance. This local model update mechanism ensures that data privacy is preserved while still allowing the model to improve with each iteration. Table 1 outlines the key phases and improvements of the proposed framework compared to existing solutions.

Accordingly, in response to the critical issue of patient privacy in modern smart technologies, our model implements state-of-the-art privacy-preserving techniques to safeguard sensitive health data as follows.

Homomorphic Encryption: The proposed framework applies homomorphic encryption to allow computations on encrypted health data without the need for decryption. This ensures that patient data remains private and secure even while being processed, preventing unauthorized access to sensitive information.

Federated Learning: To further enhance privacy, the proposed framework explores federated learning, where model training and data processing occur locally on IoMT devices such as wearables and health monitors. This approach eliminates the need to transmit patient data to central servers, thus preventing data exposure during transmission and ensuring that private health data never leaves the device.

Trusted Edge Devices: In addition, the proposed framework integrates trusted edge devices, where data is processed locally on secure, reliable devices. These devices, including wearables and smart medical devices, use privacy-preserving techniques such as federated learning and homomorphic encryption to ensure that sensitive data is never exposed or transmitted to central systems. The trustworthiness of each edge device is dynamically evaluated using a trust score $T_{k}$, which is updated based on the device’s historical performance. The trust score $T_{k}$ is adjusted by factors such as prediction accuracy and reliability, where $T_{k}^{t+1}=T_{k}^{t}+\alpha \cdot \mathrm{Success}-\beta \cdot \mathrm{Failure}$. This guarantees that only high-trust devices are involved in critical healthcare decision-making, thereby enhancing the security and reliability of the entire system.

In addition, classification metrics are evaluated to analyze the proposed framework and existing approaches. Accuracy using Equation (15) measures the correctness of the the proposed framework, indicating the proportion of correct predictions; precision denotes the proportion of correct positive predictions, focusing on the false positives, as given in Equation (16); recall using Equation (17) evaluates how many of the actual positive instances were correctly identified, focusing on the false negatives; and F1-score provides a balance between precision and recall, which is proper for imbalanced datasets where one class is significantly more frequent than the other, as given in Equation (18).

(15) $$\mathrm{Accuracy}=\frac{TP+TN}{TP+TN+FP+FN}$$

(16) $$\mathrm{Precision}=\frac{TP}{TP+FP}$$

(17) $$\mathrm{Recall}=\frac{TP}{TP+FN}$$

(18) $$F1-\mathrm{Score}=2\cdot \frac{TP}{2TP+FP+FN}$$

### 3.5. Proposed Algorithms

This section outlines the developed algorithms for healthcare applications, including data preprocessing, disability detection, and trust computation by exploring machine learning techniques for accurate classification. The proposed framework is comprised of three main algorithms. They interact with each other to achieve timely analysis and processing of health data, with efficient resource management and trust. Algorithms 1 and 2 outline the phases of data collection from health sensors, subsequent processing for disability detection, and computing trust for data integrity, and attain privacy. This involves the computation of health metrics, such as heart rate variability, motion data (MD), and speech rate (SR). After normalizing the collected data, a logistic regression technique is applied to predict disability classes, and the final class is selected based on the class with the highest probability of occurrence. Moreover, edge computing is utilized to process data locally, thereby reducing response time and improving latency for critical healthcare applications.

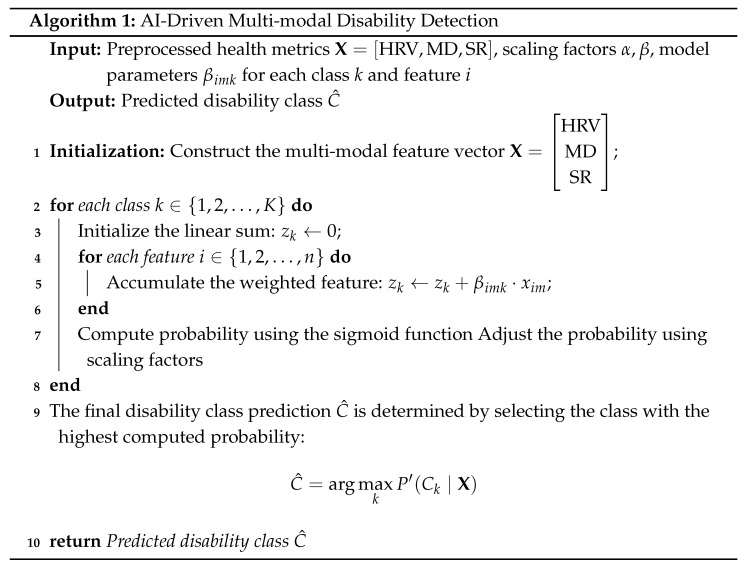

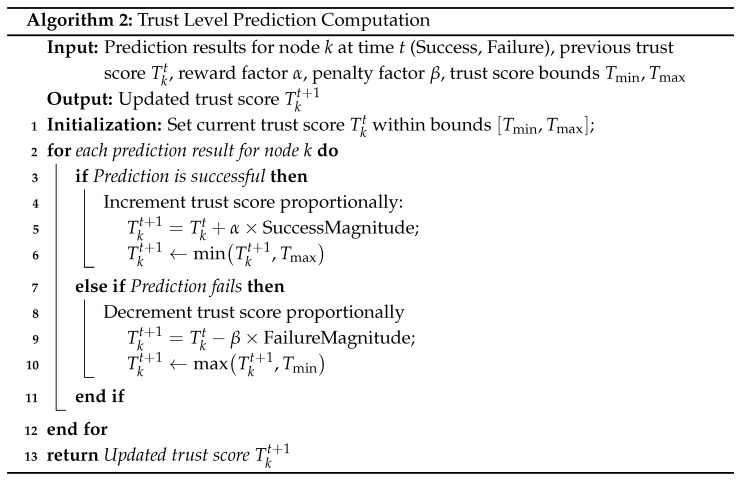





## 4. Simulation and Experimental Analysis

In this section, we present the experimental analysis and simulation environment for evaluating the proposed framework, comparing it with FDT-RPL and FC-SEEDA. The testing environment consists of 30 to 150 health sensors designed to collect and analyze continuous patient data in real-time. The deployed devices, however, are limited in terms of processing power, storage, and battery life, which influences the overall system performance. To evaluate the proposed framework, we simulate its performance using NS3 across different health sensor scenarios and sensing rate variations. We assess key performance metrics such as network throughput, the packet drop ratio, response time, latency, and availability to understand how the system performs under different operating conditions. We used the WISDM [38] and Speech Emotion Recognition [39] datasets to train and validate the model’s disability detection capabilities. These datasets provide the foundation for measuring the health metrics necessary for assessing patient conditions. We utilized statistical methods to analyze these datasets and ensure the consistency and accuracy of the disability detection process within the framework. Table 2 illustrates the values of the simulation parameters used in the evaluation of our proposed framework.

### Discussion

**Figure 3:**
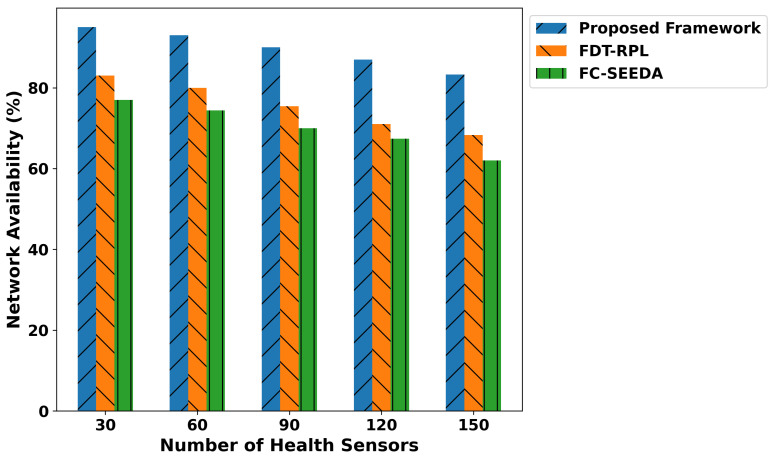
Performance of the proposed framework for network availability under varying health sensors.

**Figure 4:**
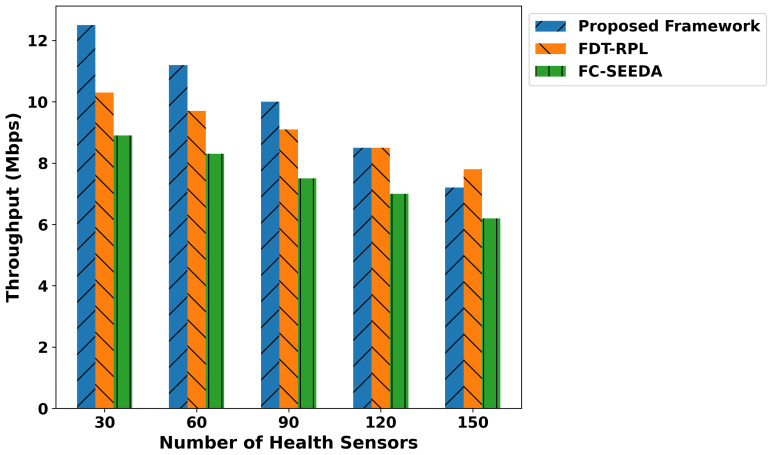
Performance of the proposed framework for network throughput under varying health sensors.

In Figure 3, the proposed framework illustrates the significant improvement in network availability compared to existing approaches. Based on the experimental analysis, the proposed framework demonstrated improvements of 57.6% and 63.2% compared to FDT-RPL and FC-SEEDA, respectively, across varying numbers of health devices. This efficiency is due to trust-driven data routing in the proposed model, which selects more reliable routes for transmitting medical data. In addition, the incorporation of edge-level computation not only decreases the computational load on health devices but also provides more reliable distributed communication systems with efficient detections of disabilities in patients’ bodies promptly. Furthermore, detecting malicious threats in the healthcare data prevents unnecessary retransmissions and conserves devices’ energy by optimizing the smart diagnostic system. In Figure 4, network throughput performance is measured for the proposed framework and existing work. It was observed that the proposed framework consistently provides a higher network throughput by an average of 48% and 59.2% over varying health devices. This is attributed to providing a more robust and fault-tolerant approach to managing medical records on constrained resources. The proposed trust-aware routing in real-time healthcare applications dynamically selects the most consistent peer connections and congestion-free paths, utilizing multi-feature selection to support this process. In addition, the exploration of logistic regression provides intelligent learning with more reliable predictions than the single classifier, thereby leading to a rapid and accurate decision-making system. In the proposed framework, an AI-driven approach plays a key role in detecting disability; however, the integration of edge computing explicitly reduces the additional congestion on medical devices, and by performing disease classification locally, it significantly enhances the network throughput for crucial healthcare operations and demanded services.

**Figure 5:**
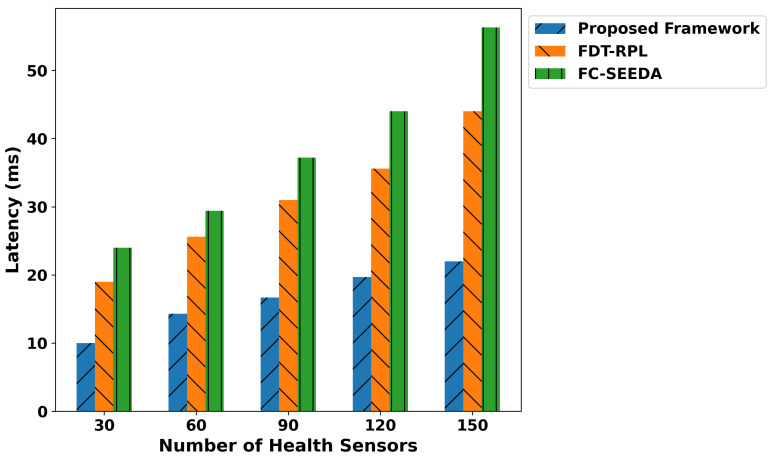
Performance of the proposed framework for network throughput under varying health sensors.

**Figure 6:**
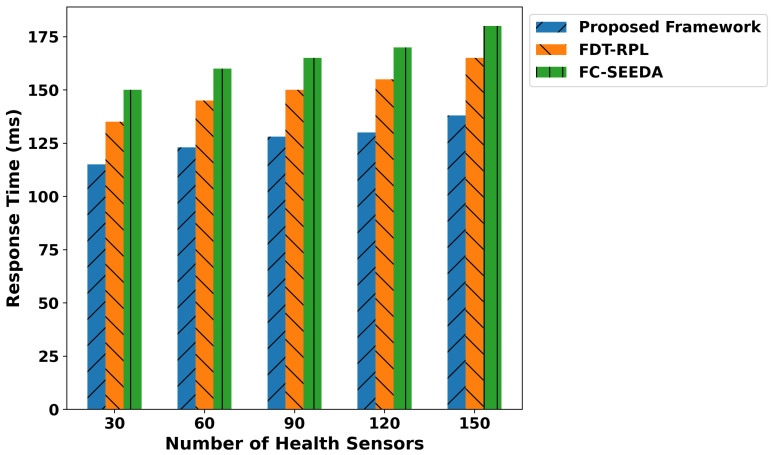
Performance of the proposed framework for network throughput under varying health sensors.

Figure 5 demonstrates the performance analysis of the proposed framework and related studies in terms of latency. It was observed that the proposed framework significantly reduces data latency by an average of 40.3% and 56% compared to FDT-RPL and FC-SEEDA, respectively. This is due to the integration of a trust-aware and lightweight strategy when sending health records to the cloud server for disease analysis. It avoids unreliable or malicious devices, thereby decreasing additional delays and maintaining updated routes for the forwarding of medical data. By exploring lightweight and robust edge-driven processing for disability classification using a collection of real-time patient data, the proposed framework enables faster responses to connected devices for medical treatments. In addition, healthcare applications facilitate optimal communication through the combination of trust score computations and reduce malicious traffic with minimal data delays. Moreover, the proposed framework can process the most urgent data and prioritize the processing of critical health data, resulting in the timely delivery of data to the requested devices in medical systems. In Figure 6, the performance evaluation of the proposed framework is illustrated through a comparison of the IFDT-RPL and FC-SEEDA approaches. The results analysis demonstrated an average improvement of 47.4% and 56.7% in performance for the proposed framework compared to existing approaches. The intelligence of edge-level processing enables rapid processing and reduces response time by classifying patient movement patterns based on disability-related medical signals. It enables the timely detection of abnormalities in patients’ bodies, allowing medical experts to provide rapid treatment with minimal communication delays. In addition, the trust-based routing decision of the proposed framework ensures that consistent devices are selected for intermediate points in local-level processing, thereby avoiding bottlenecks in healthcare forwarding routes. It enables rapid response for critical health conditions and leads to appropriate actions to address patient diagnostic abnormalities. By incorporating fault-tolerant parameters into the proposed framework and considering fresh readings from health sensors, the performance significantly improves for continuous disability monitoring and emergency healthcare services.

**Figure 7:**
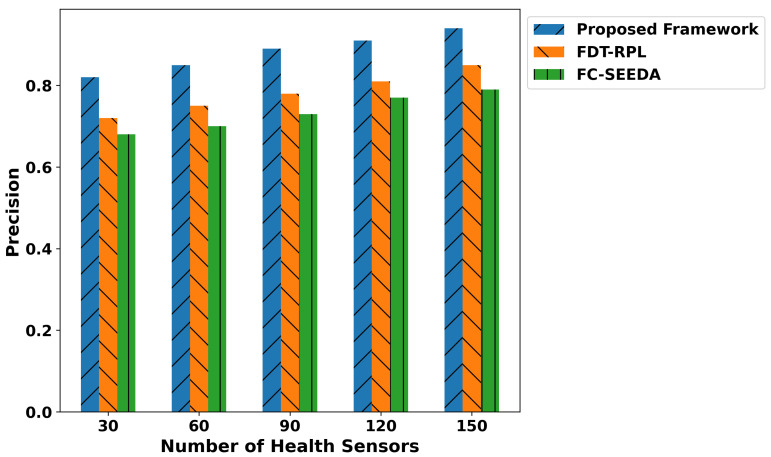
Performance of precision for proposed framework, FDT-RPL, and FC-SEEDA.

**Figure 8:**
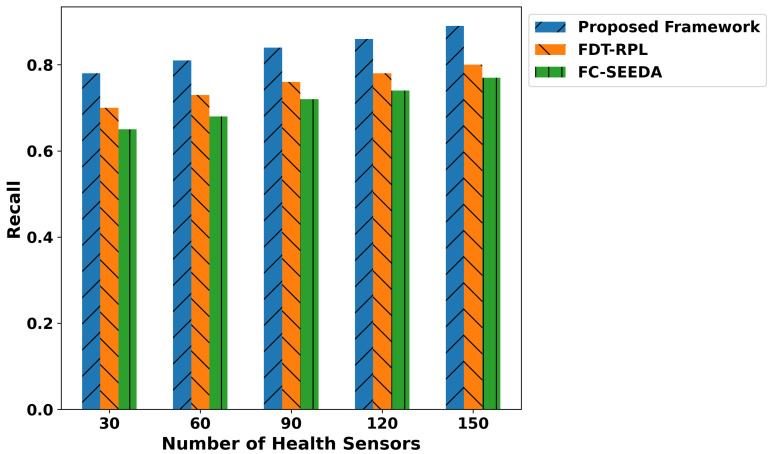
Performance of recall for proposed framework, FDT-RPL, and FC-SEEDA.

**Figure 9:**
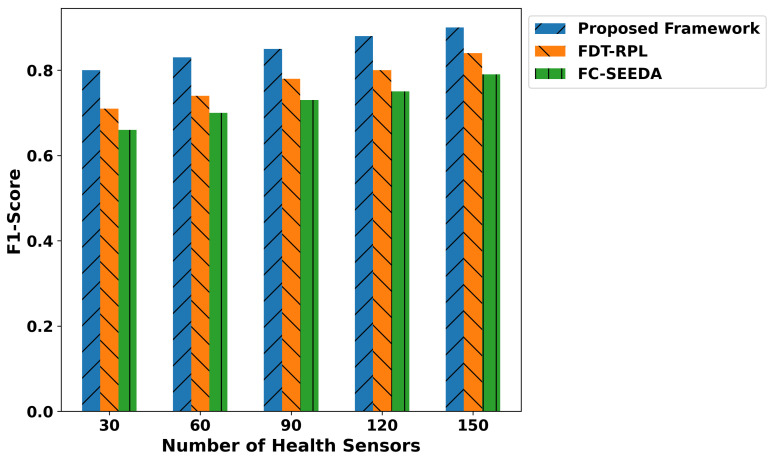
Performance of F1-score for proposed framework, FDT-RPL, and FC-SEEDA.

**Figure 10:**
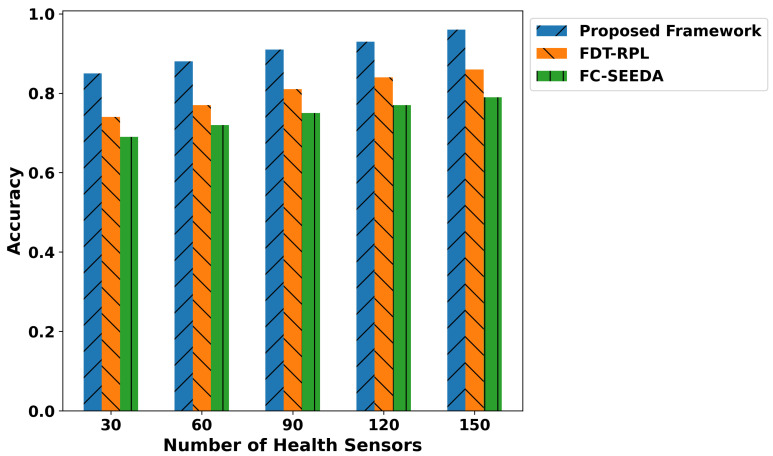
Performance of accuracy for proposed framework, FDT-RPL, and FC-SEEDA.

The proposed framework improves the classification metrics in terms of precision, recall, F1-score, and accuracy as depicted in Figure 7, Figure 8, Figure 9 and Figure 10. Integrating logistic regression and an edge-level trust-driven approach reduces the congestion and unreliability of health devices in decision-making policies. Moreover, unlike traditional models, which primarily rely on a single factor or raw sensor readings, the proposed framework explores secure and more authentic routes for forwarding health records using multi-feature-based disability classifications. Moreover, the logistic regression model was trained using updated features, incorporating the trust score of devices and constraint thresholds, thereby improving precision and recall for real-time disability detection in medical applications. In addition, the proposed framework enhances the F1-score by integrating trust-driven data filtering, secure forwarders, and multi-feature-based logistic regression classification. It identifies malicious data by exploring the trusted score, and only prioritized health records are transmitted for processing to detect abnormalities.

**Figure 11:**
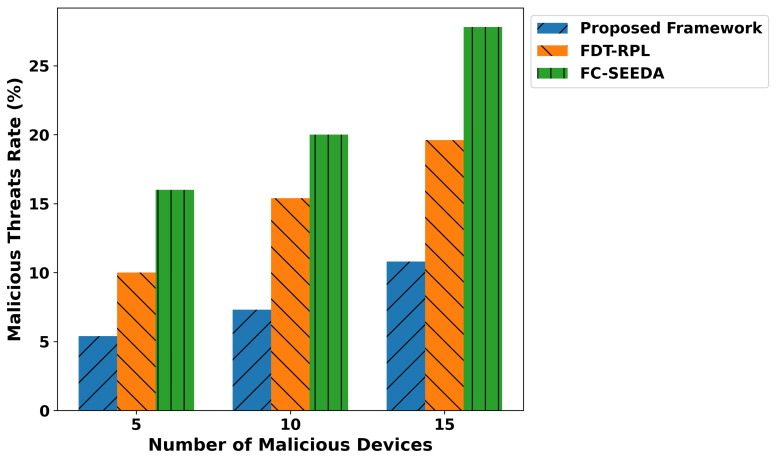
Performance of malicious threats for proposed framework, FDT-RPL, and FC-SEEDA.

**Figure 12:**
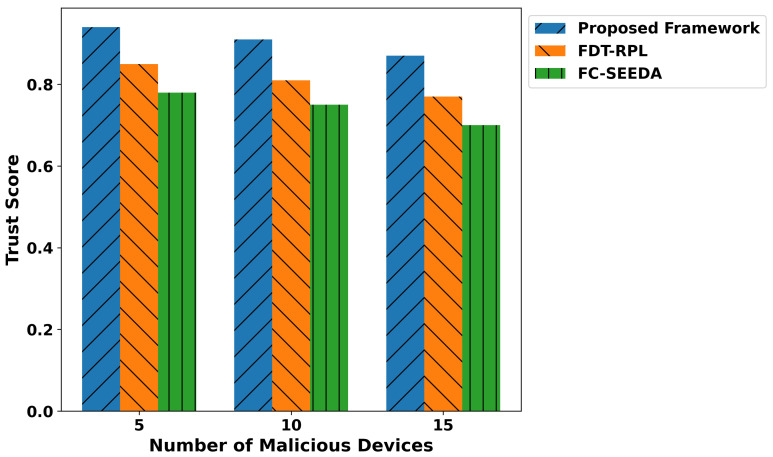
Performance of trust score for proposed framework, FDT-RPL, and FC-SEEDA.

**Figure 13:**
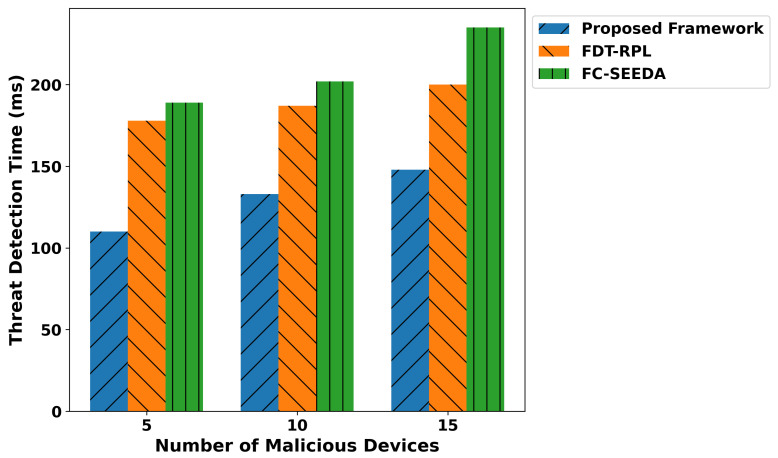
Performance of threat detection time for proposed framework, FDT-RPL, and FC-SEEDA.

Figure 11 illustrates the performance of the proposed framework and existing studies regarding malicious threats under varying malicious devices. It ensures the privacy of critical medical data through lightweight, secure computation, providing a proactive and reliable routing approach for real-time disability detection in healthcare applications. Based on the experiments, it was observed that the proposed framework reduced the malicious threats by an average of 40% and 49.3%, respectively, compared to IFDT-RPL and FC-SEEDA. It ensures that the IoMT system is more suited for real-time healthcare applications that require security and intelligence for real-time network services. Additionally, the edges play a crucial role in identifying authentic devices while transmitting real-time health records to a cloud server for further processing. Only verified devices are permitted to be part of the IoMT system. It reduces false traffic on communication links and increases the trustworthiness level for smart and unpredictable technologies. Figure 12 illustrates the performance evaluation of the proposed framework in comparison with existing solutions, specifically in terms of the trust score against varying malicious devices. Based on the statistical analysis, it was observed that the proposed framework improved the trust level, security, and reliability, especially in real-time disability detection healthcare applications. By incorporating a trust evaluation mechanism based on multiple features, the proposed TRDD-SRF identifies unauthentic devices and isolates them in separate tables for logging and blocking the constructed data route, thereby preventing the forwarding of health operations. It ensured secure data transmission with lightweight edge-level processing and integrity checks, thereby improving the trust level by an average of 46.2% and 53.4% compared to IFDT-RPL and FC-SEEDA. The trusted computation not only facilitates the timely and practical response of health services to connected devices but also ensures the reliable delivery of medical-related decisions based on recent sensor-observed patient data. The proposed framework’s adopted mechanisms enhance the security level of generating alert messages on time and protect sensitive health information from risks, thereby providing assistive healthcare applications. Over varying faulty devices, the performance evaluation of the proposed framework is given in Figure 13. It was discovered that the proposed framework improves the detection time for malicious threats by an average of 39% and 48% compared to existing studies. It results from developing mutual collaboration and a reliable connection by integrating robust, trusted devices and protected healthcare data at network edges. It significantly reduces the detection time for malicious threats by leveraging lightweight computation for trust evaluation and device behavior. In addition, the proposed framework’s cross-layer approach and device feedback enable it to rapidly identify abnormal activities in a real-time, disability-focused healthcare system. It improves the identification process of malicious devices, leads to a more robust solution for managing disability-related health records, and enhances diagnostic accuracy for real-time healthcare services.

## 5. Conclusions

Healthcare applications, combined with medical devices, have advanced in the real-time monitoring of data acquisition from the physical environment. They are interconnected with future wireless technologies to observe patients’ crucial data and forward it to processing systems. Such advancements not only facilitate automated analysis but also provide efficient strategies for decision-making processes and disease diagnosis. Moreover, due to the limited constraints and the adaptive nature of the medical environment, most existing work faces research challenges related to accurate data analysis, resource management, and privacy threats. Furthermore, the medical devices should interact with each other with the support of a trusted system to enhance the reliability of the health data against malicious computing. To address these challenges, we proposed an AI-based autonomous framework leveraging edge computing to enhance disability detection with timely response while reducing the risk of manipulation threats. The framework intelligently manages healthcare application resources by combining multi-modal data using logistic regression and efficiently processing large-scale medical data. It is specifically tailored for constrained devices with limited processing and storage capacities, ensuring the accurate analysis of extensive medical records. Incorporating trust into the framework further ensures consistent and reliable disability detection, fostering secure interaction among devices for medical operations. However, the current evaluation relies on publicly available healthcare datasets, which may limit generalization to diverse patient populations. As part of future work, we plan to incorporate clinical validation and utilize transfer learning to enhance adaptability and detection accuracy, and integrate robust security mechanisms.

## Figures and Tables

**Table 1:** Phases of the proposed framework and key improvements over existing approaches.

Phase	Enhanced Functionality and Contribution
xHealthcare Data Collection and Preprocessing	Integrates multi-sensor signals (HRV, motion, speech), enhancing health representation. Applies normalization to reduce sensor heterogeneity, a factor often ignored in previous models. Extracts comprehensive physiological and behavioral metrics for robust input features. Existing work limitation: Previous systems used limited features or single-modal data.
AI-Driven Early Disability Detection	Uses an interpretable logistic model for disability classification across multiple factors. Employs a structured feature vector, improving explainability and deployment readiness. Supports scalability to multiple classes using soft decision boundaries. Existing work limitation: Most studies rely on binary or isolated symptom detection.
Lightweight Trust Level Prediction Computation	Introduces a dynamic trust score that adapts to prediction performance. Incorporates reward (α) and penalty (β) factors for real-time reliability tuning. Enables continuous learning and accountability tracking. Existing work limitation: Trust modeling is often static or manually tuned.

**Table 2:** Simulation Parameters.

Parameter	Value
Initial energy	2 j
Health Sensors	30, 60, 90, 120, 150
Number of edges	10
Size of packets	512 bits
Nodes deployment	Random
Sensing radius	3 m
Data packets	5 to 50
Edge Devices	20
Malicious nodes	30
MAC layer	IEEE 802.11b
Analyzing Scenarios	Varying health devices and sensing rates
Simulations run	50

## Data Availability

All data is available in the manuscript.
